# Human papillomavirus infection in women with and without cervical cancer in Karachi, Pakistan

**DOI:** 10.1038/sj.bjc.6605664

**Published:** 2010-04-20

**Authors:** S A Raza, S Franceschi, S Pallardy, F R Malik, B I Avan, A Zafar, S H Ali, S Pervez, S Serajuddaula, P J F Snijders, F J van Kemenade, C J L M Meijer, S Shershah, G M Clifford

**Affiliations:** 1 Department of Surgery, The Aga Khan University, Stadium Road, Karachi 74800, Pakistan; 2 International Agency for Research on Cancer, 150 cours Albert Thomas, 69372 Lyon Cedex 08, France; 3 Department of Gynecology and Obstetrics, Sindh Government Qatar Hospital, Sector 8L, Orangi Town, Karachi 75800, Pakistan; 4 Immpact, School of Medicine and Dentistry, University of Aberdeen, 3rd Floor Polwarth Building, Foresterhill, Aberdeen AB25 2ZD UK; 5 Department of Pathology and Microbiology, The Aga Khan University, Stadium Road, Karachi 74800, Pakistan; 6 Department of Biological and Biomedical Sciences, The Aga Khan University, Stadium Road, Karachi 74800, Pakistan; 7 Department of Pathology and Microbiology, Ziauddin Medical University, 4/B Shahrah-e-Ghalib, Block 6, Clifton, Karachi 75600, Pakistan; 8 Department of Pathology, Vrije University Medical Center, Postbus 7057, 1007 MB Amsterdam, the Netherlands

**Keywords:** HPV, prevalence, cervical cancer, Pakistan

## Abstract

**Background:**

No data exist on the population prevalence of, or risk factors for, human papillomavirus (HPV) infection in predominantly Muslim countries in Asia.

**Methods:**

Cervical specimens were obtained from 899 married women aged 15–59 years from the general population of Karachi, Pakistan and from 91 locally diagnosed invasive cervical cancers (ICCs). HPV was detected using a GP5+/6+ PCR-based assay.

**Results:**

The prevalence of HPV in the general population was 2.8%, with no evidence of higher HPV prevalence in young women. The positivity of HPV was associated with women's lifetime number of sexual partners, but particularly with the age difference between spouses and other husbands’ characteristics, such as extramarital sexual relationships and regular absence from home. The HPV16/18 accounted for 24 and 88% of HPV-positive women in the general population and ICC, respectively.

**Conclusion:**

Cervical cancer prevention policies should take into account the low HPV prevalence and low acceptability of gynaecological examination in this population.

To date, there are no data on the population prevalence of, or risk factors for, human papillomavirus (HPV) infection in predominantly Muslim countries in Asia, where sexual mores differ from many other world populations (Wellings *et al*, 2006). These data are essential to assess the potential relevance of HPV vaccination and HPV test-based screening to invasive cervical cancer (ICC) prevention in the region, as well as to identify any changes in risk occurring in young generations. Thus, a study of women with and without cervical cancer was carried out in Karachi, Pakistan, according to the standardised protocol of the International Agency for Research on Cancer (IARC) HPV Prevalence Surveys (Clifford *et al*, 2005), which was approved by both the IARC and local ethical review committees.

## Methods

A total of 3882 married women aged 15–59 years living in Orangi, a densely populated suburb of Karachi, were visited at their homes and invited to join the study, with the aim to enrol ∼100 women in each 5-year age group. Participation rates were 24.1, 25.7, 25.1 and 23.6% among women aged 15–24, 25–34, 35–44 and 45–59 years, respectively. All participants signed an informed consent form and were administered a questionnaire. In all, 915 participants came to the study clinic located in the Sindh Government Qatar Hospital, where a sample of exfoliated cervical cells was collected and placed into PreservCyt media (Hologic, Marlborough, MA, USA) for HPV testing and liquid-based cytology.

In parallel, formalin-fixed tumour biopsies were retrieved from women presenting with histologically confirmed ICC between 2004 and 2008 to the Ziauddin and Aga Khan University Hospitals, Karachi. After exclusion of 40 biopsies that were *β*-globin negative and/or without histological evidence of tumour, 91 ICCs remained (79 squamous cell, 3 adeno, 4 small cell and 5 other or unspecified carcinomas).

Liquid-based cytology and HPV testing were carried out at the Vrije University, Amsterdam, the Netherlands. A general primer GP5+/6+-mediated PCR was used for the detection of 44 genital HPV types (Jacobs *et al*, 2000). Subsequent HPV typing was carried out by reverse-line blot hybridisation of PCR products (van den Brule *et al*, 2002). Odds ratios (ORs) for HPV positivity were calculated by unconditional logistic regression adjusted for age, with OR trends assessed by considering categories as continuous variables.

## Results

Among 899 women from the general population with valid cytology and HPV results, HPV prevalence was 2.8% (Table 1). Cervical abnormalities were diagnosed in 2.4% of women, of whom 27.3% were HPV-positive. They included 19 atypical squamous cells of undetermined significance (4 HPV-positive), 1 low-grade squamous intraepithelial lesion (HPV16/66-positive) and 2 high-grade squamous intraepithelial lesions (1 HPV-negative; 1 HPV16-positive, later revealed to be ICC). HPV16 was confirmed as the most common type among women with both normal (0.5%) (Clifford *et al*, 2005) and abnormal (9.1%) cytology. HPV16 was also the predominant HPV type (75.8%) in ICC, followed by HPV18 (6.6%) and 45 (4.4%) (Table 1).

Having ⩾2 lifetime sexual partners was reported by 5.6% of women, and was significantly associated with HPV positivity (OR=3.36; 95% confidence interval (CI): 1.08–10.41), as was being a working woman (OR=3.01; 95% CI: 1.26–7.21) (Table 2). In addition, HPV infection was strongly associated with husbands’ characteristics, such as extramarital sexual relationships (OR=4.40; 95% CI: 1.83–10.56), absence from home >7 nights per month (OR=4.84; 95% CI: 1.30–17.95) and a ⩾10 year age difference between spouses (OR=6.88; 95% CI: 1.48–31.94) (Table 2). Husbands’ characteristics were correlated with each other, but associations were unaffected by adjustment for women's lifetime number of sexual partners.

The positivity of HPV did not vary significantly by age (Table 2). Neither were there any significant associations with education level, marital status, number of full-term pregnancies, age at sexual debut (Table 2), language/ethnic group (87.5% Urdu speakers), birth outside Karachi (44.9%), use of condom (19.7%), hormonal contraceptives (16.7%) and intrauterine device (7.2%), tubal ligation (5.2%), history of spontaneous (35.2%) or induced (11.5%) abortions, smoking (1.7%) or previous Pap smear (1.1%) (data not shown).

## Discussion

This study disclosed a very low burden of HPV infection in the general female population of Karachi, considerably lower than that found using similar protocols in nearby India (17%) (Franceschi *et al*, 2005), China (15–18%) (Dai *et al*, 2006; Li *et al*, 2006; Wu *et al*, 2007) and Nepal (9%) (Sherpa *et al*, 2010), and >10-fold lower than in sub-Saharan Africa (e.g., 51% in Guinea, also a predominantly Muslim country) (Keita *et al*, 2009). There was no evidence of higher prevalence in younger women, confirming the flat age-specific curve seen in other low-resource countries in Asia and Africa (Franceschi *et al*, 2006), although at much lower HPV prevalence. An HPV prevalence of 27.3% in cervical abnormalities is also low compared with high-resource settings (IARC, 2005), highlighting problems in specificity of even gold-standard cytology in settings of very low HPV prevalence.

The HPV16 accounted for three-quarters of ICC in Pakistan, confirming the high prevalence of HPV16 observed in ICC from the Indian subcontinent compared with other world regions (Franceschi *et al*, 2003; Basu *et al*, 2009; Gheit *et al*, 2009). Indeed, our data would suggest that the predominance of HPV16 over other high-risk types might be even greater in settings of low HPV exposure.

Major study strengths include a large population-based sample, use of a standardised and well-validated HPV test allowing comparisons with similar studies around the world (Clifford *et al*, 2005) and with a concurrent series of ICC from the same area. The main limitation, which was nevertheless an important finding in itself, was the low participation rate, for which the principal reasons were lack of husband's permission and/or lack of appreciation of screening in the absence of symptoms. As HPV infection is asymptomatic, it is unlikely that participation was highly correlated to HPV positivity. However, hesitancy to undergo gynaecological examination would be a strong obstacle to any future cervical cancer screening efforts in Pakistan (Imam *et al*, 2008). Furthermore, vaginal examination was acceptable only to married women, as in similar surveys in Asia (Franceschi *et al*, 2005; Dai *et al*, 2006; Sherpa *et al*, 2010). Nevertheless, 98.2% of women reported an age at sexual debut concurrent with, or just after, age at marriage, suggesting that marriage may be a good proxy of sexual debut in this population.

In conclusion, the low HPV prevalence in this study is consistent with the low ICC risk estimate from Karachi (7.5/100 000 cases annually; rarer than the breast, mouth, ovary and oesophagus) (Curado *et al*, 2007) and other predominantly Muslim countries in Asia (Curado *et al*, 2007) and, reassuringly, there is no evidence that HPV prevalence is higher in young generations of urban women. However, 80% of the ICC that do develop in this population (of which one was newly diagnosed by our study) are attributable to HPV16/18. These findings should be taken into account when considering the cost-effectiveness of ICC prevention modalities in a population in which the burden of HPV and other sexually transmitted infections (Zaheer *et al*, 2009) seems low, but the burden of many other chronic infectious diseases (e.g., tuberculosis (Hasan *et al*, 2009) and hepatitis C virus (Ahmad, 2004)) is high.

## Figures and Tables

**Table 1:** Prevalence of HPV types in 899 women from the general population and 91 women with ICC in Karachi, Pakistan

	**General population**							
	**Normal cytology**			**Abnormal cytology**			**All (%)**	**ICC** **All (%)**
**HPV type**	**Sing.**	**Mult.**	**Total (%)**	**Sing.**	**Mult.**	**Total (%)**		
*N*			877			22	899	91
HPV+	16	3	19 (2.2)	3^a^	3^b^	6 (27.3)	25 (2.8)	83^c^ (92.2)
								
*High-risk*								
16	3	1	4 (0.5)	1^a^	1^b^	2 (9.1)	6 (0.7)	69 (75.8)
18	—	—	—	—	—	—	—	6 (6.6)
33	—	—	—	—	—	—	—	1 (1.1)
35	2	—	2 (0.2)	—	—	—	2 (0.2)	—
45	—	—	—	—	1	1 (4.5)	1 (0.1)	4 (4.4)
51	1	1	2 (0.2)	—	—	—	2 (0.2)	—
56	2	—	2 (0.2)	—	—	—	2 (0.2)	2 (2.2)
59	1	—	1 (0.1)	—	—	—	1 (0.1)	1 (1.1)
68	—	—	—	—	1	1 (4.5)	1 (0.1)	—
Any	9	2	11 (1.3)	1	3	4 (18.2)	15 (1.7)	81 (89.0)
								
*Low-risk*								
6	2	—	2 (0.2)	1	—	1 (4.5)	3 (0.3)	—
40	—	1	1 (0.1)	—	—	—	1 (0.1)	—
42	2	2	4 (0.5)	—	—	—	4 (0.4)	1 (1.1)^d^
43	—	—	—	—	1	1 (4.5)	1 (0.1)	—
54	1	—	1 (0.1)	—	—	—	1 (0.1)	—
66	—	—	—	—	1^b^	1 (4.5)	1 (0.1)	1 (1.1)
67	—	1	1 (0.1)	—	—	—	1 (0.1)	—
69	—	—	—	—	—	—	—	1 (1.1)
70	—	2	2 (0.2)	—	—	—	2 (0.2)	—
84	1	—	1 (0.1)	—	—	—	1 (0.1)	—
CP6108	1	—	1 (0.1)	—	1	1 (4.5)	2 (0.2)	—
Any	7	3	10 (1.1)	1	3	4 (18.2)	14 (1.6)	3 (3.3)
*X*	—	—	—	1	—	1 (4.5)	1 (0.1)	1 (1.1)

Abbreviations: HPV=human papillomavirus; ICC=invasive cervical cancer; mult.=multiple; sing.=single.

a Including one woman with high-grade squamous intraepithelial lesion or ICC.

b Including one woman with low-grade squamous intraepithelial lesion.

c Including three multiple infections (16/18, 16/18/66, 16/69), which are each counted separately.

d One ICC was positive for a low-risk type only.

**Table 2:** ORs for HPV positivity and corresponding 95% CIs according to selected characteristics among 899 women in Karachi, Pakistan

		**HPV-positive**			
**Characteristic**	***N* women** a	***N***	**%**	**OR** ^**b**^	**95% CI**
*Age (years)*					
<25	199	4	2.01	1	—
25–34	235	7	2.98	1.50	0.43–5.19
35–44	246	6	2.44	1.22	0.34–4.38
⩾45	219	8	3.65	1.85	0.55–6.24
*χ*^*2*^_1_ for trend				0.71	*P*=0.4
					
*Education level*					
Secondary or higher	209	3	1.44	1	—
Primary or middle	319	6	1.88	1.35	0.33–5.49
Illiterate	371	16	4.31	3.22	0.88–11.84
*χ*^*2*^_1_ for trend				4.31	*P*=0.04
					
*Occupation*					
Housewife	774	17	2.20	1	—
Employed	125	8	6.40	3.01	1.26–7.21
					
*Marital status*					
Married, husband has one wife	784	20	2.55	1	—
Married, husband has more than one wife	44	2	4.55	1.69	0.38–7.51
Separated or divorced	26	2	7.69	3.36	0.74–15.36
Widow	45	1	2.22	0.70	0.09–5.61
					
*Number of full-term pregnancies*					
0	110	3	2.73	1.24	0.31–4.98
1–3	302	7	2.32	1	—
⩾4	480	15	3.13	1.21	0.39–3.78
*χ*^*2*^_1_ for trend				0	*P*=1
					
*Lifetime number of sexual partners*					
1	845	20	2.37	1	—
⩾2	50	4	8.00	3.36	1.08–10.41
					
*Age at sexual debut (years)*					
⩽15	176	7	3.98	1	—
16–18	333	6	1.80	0.46	0.15–1.41
⩾19	389	12	3.08	0.83	0.30–2.29
*χ*^*2*^_1_ for trend				0.01	*P*=0.9
					
*Age difference with husband* c					
0–4 years	269	2	0.74	1	—
5–9 years	366	10	2.73	3.66	0.79–16.87
⩾10 years	193	10	5.18	6.88	1.48–31.94
*χ*^*2*^_1_ for trend				7.25	*P*=0.007
					
*Husband's extramarital sexual relationships* c					
No	693	12	1.73	1	—
Yes	134	10	7.46	4.40	1.83–10.56
					
*Husband away from home* >*7 nights per month*^c^					
No	801	19	2.37	1	—
Yes	26	3	11.54	4.84	1.30–17.95

Abbreviations: CI=confidence interval; HPV=human papillomavirus; OR=odds ratio.

a Some figures do not add up to the total because of few missing values.

b Adjusted for age.

c Restricted to currently married women only (*N*=828).
